# Defining Mechanisms of C_3_ to CAM Photosynthesis Transition toward Enhancing Crop Stress Resilience

**DOI:** 10.3390/ijms241713072

**Published:** 2023-08-22

**Authors:** Bowen Tan, Sixue Chen

**Affiliations:** Department of Biology, University of Mississippi, Oxford, MS 38677, USA; btan@go.olemiss.edu

**Keywords:** Crassulacean acid metabolism, C_3_ to CAM transition, facultative CAM, photosynthesis, climate change, water-use efficiency, CAM engineering

## Abstract

Global climate change and population growth are persistently posing threats to natural resources (e.g., freshwater) and agricultural production. Crassulacean acid metabolism (CAM) evolved from C_3_ photosynthesis as an adaptive form of photosynthesis in hot and arid regions. It features the nocturnal opening of stomata for CO_2_ assimilation, diurnal closure of stomata for water conservation, and high water-use efficiency. To cope with global climate challenges, the CAM mechanism has attracted renewed attention. Facultative CAM is a specialized form of CAM that normally employs C_3_ or C_4_ photosynthesis but can shift to CAM under stress conditions. It not only serves as a model for studying the molecular mechanisms underlying the CAM evolution, but also provides a plausible solution for creating stress-resilient crops with facultative CAM traits. This review mainly discusses the recent research effort in defining the C_3_ to CAM transition of facultative CAM plants, and highlights challenges and future directions in this important research area with great application potential.

## 1. Introduction

Drastic climate change over the past decades can be reflected by the alternation in atmospheric CO_2_ levels, tropospheric ozone concentrations, and other environmental indicators [1]. Climate change is not only affecting ecosystems, but also agriculture, food production, land, and water resources [2]. Arid or semi-arid land accounts for around 41% of the total surface on Earth, and it is expanding [3]. In 2035, global desertification is projected to be 65% of the total land surface in the subtropical regions [4]. With the rapid growth of the human population, the demand for food is increasing, and it is anticipated to surge by 70%. The current rate of global crop productivity only increases by ~2% per year, which cannot meet the demand for food [5]. To worsen the situation, the global decrease in freshwater from 1980 to 2015 has caused a 20.6% and 39.3% yield reduction in wheat and maize, respectively [6].

**Figure 1:**
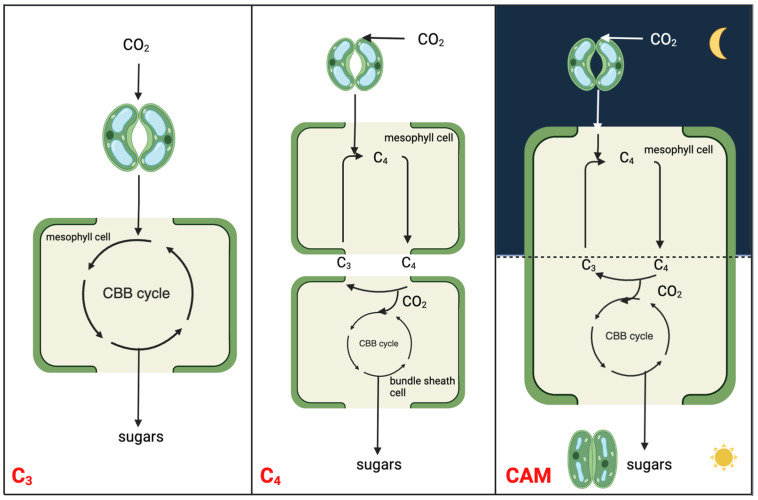
Simplified schematic to illustrate the molecular relationships and distinctions among C_3_, C_4_, and CAM photosynthesis mechanisms. The CBB cycle is the abbreviation of Calvin-Benson-Bassham cycle, which is also known as Calvin cycle.

Photosynthesis, a pivotal biological process essential to all life, provides food and most of our energy resources [7]. There are three major modes of photosynthesis in vascular plants to assimilate atmospheric CO_2_: C_3_, C_4,_ and Crassulacean acid metabolism (CAM) [8,9] (Figure 1). CAM photosynthesis has evolved independently multiple times from C_3_ as a photosynthetic adaptation to cope with the decreasing atmospheric CO_2_ levels ~20 million years ago [10]. CAM plants are commonly found in harsh environments such as arid and semi-arid regions [11]. Other than those water-limited regions, CAM plants also inhabit the aquatic environment. With the release of the genome and transcriptome of an underwater CAM plant *Isoetes taiwanensis* [12], differences in the recruitment of phosphoenolpyruvate (PEP) carboxylase (PEPC) and core CAM pathway gene expression between aquatic and terrestrial plants demonstrate a different route of CAM evolution.

CAM is a carbon concentrating mechanism, with the capability of assimilating CO_2_ initially at night using PEPC in the cytosol, leading to the formation of a four-carbon malate, which is then stored in the vacuole [13,14]. The three-carbon acceptor in this reaction is PEP, which is replenished by the glycolytic breakdown of carbohydrate storage in the form of starch or other sugars. Unlike spatial decoupling of carboxylation and decarboxylation in C_4_ photosynthesis, CAM photosynthesis separates these two processes in a temporal manner to shield ribulose-1,5-bisphosphate carboxylase-oxygenase (RuBisCO) from the oxygenase activity, minimizing photorespiration (Figure 1). CAM plants conduct gas exchange predominantly at night when the air temperature is low, thereby having a lower water loss by an order of magnitude than it would be during the day [15]. As such, CAM plants have water-use efficiency (WUE) several-fold higher than those of C_3_ and C_4_ plants under comparable conditions [16]. High WUE, together with enhanced heat and drought tolerance, drives the basic and applied research on CAM toward crop CAM engineering/bio-design. The typical diel cycle of CAM entails four phases: (I) nocturnal atmospheric CO_2_ fixation by PEPC and malic acid storage in the vacuole; (II) RuBisCO activation just after dawn when, for a brief period, CO_2_ is fixed by both PEPC and RuBisCO; (III) stored malate decarboxylated to CO_2_, which is fixed by RuBisCO; (IV) the end of the light period when stomata reopen driven by the depletion of malate pool [13,17].

CAM plants normally exhibit the following features: the diurnal fluctuation of malic acids (accumulation during the night period and dissipation during the day); reciprocal diurnal fluctuation of storage carbohydrates such as starch, polyglucans, or soluble hexoses; a high level of PEPC and an active decarboxylase; large storage vacuoles that are in the same cells with chloroplasts; water-limitation related traits, such as dense trichomes, leaf succulence, and waxy cuticles [1]; and nocturnal net CO_2_ uptake, which exhibits an inverse pattern of stomatal movement [18]. In the course of evolution, an intermediate CAM mechanism called facultative CAM arose. Plants conducting facultative CAM demonstrate the optional use of CAM under stress conditions, while remaining the use of C_3_ or C_4_ photosynthesis under normal conditions [19,20,21,22,23].

**Figure 2:**
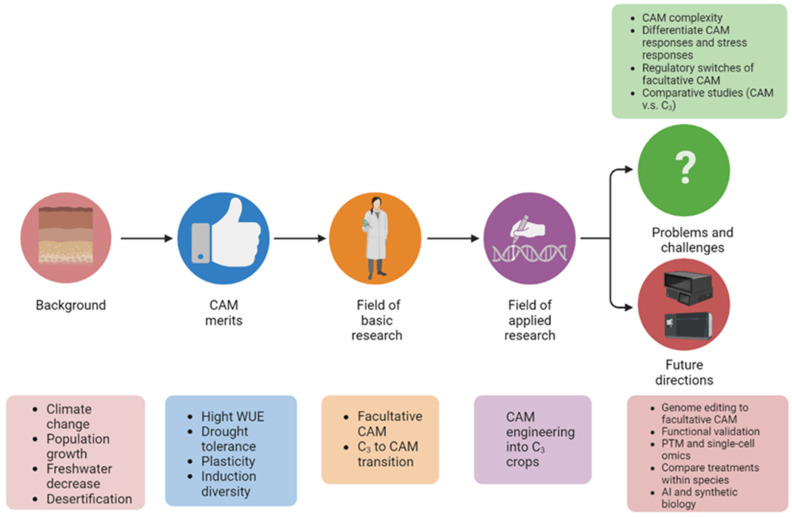
Graphical summary of the significance of studying C_3_ to CAM transition of facultative CAM plants. The abbreviations used: C_3_, C_3_ photosynthesis; CAM, Crassulacean acid metabolism; WUE, water-use efficiency; and PTM, post-translational modification.

Given the characteristics of facultative CAM photosynthesis and climate change urgency, unraveling the molecular mechanisms underlying the C_3_ to CAM transition has attracted growing interest. However, there is a lack of review on facultative CAM and especially C_3_ to CAM transition. Herein we summarize recent advances in facultative C_3_ to CAM transition, discuss current problems and challenges, and highlight future research directions (Figure 2).

## 2. The Plasticity of CAM Is Best Represented in Facultative CAM

A remarkable hallmark of CAM plants is their considerable plasticity in expressing the four phases of CAM, while keeping the C_3_ cycle fully functional [24]. Environmental factors such as light intensity, relative humidity, water availability [25], and developmental stages [16] affect the degree and duration of CAM expression [9]. CAM is highly plastic and can operate in different modes: (1) obligate/constitutive CAM or strong CAM, with high nocturnal acid accumulation (ΔH^+^) and CO_2_ fixation; (2) CAM-cycling, with daytime CO_2_ fixation like C_3_ and nocturnal fixation of CO_2_ from respiration; (3) CAM idling, with stomata closed all the time and CAM fixation of CO_2_ only from respiration; (4) facultative/inducible CAM, with C_3_ mode of CO_2_ fixation and zero ΔH^+^ in the non-stressed state, and small nocturnal CO_2_ fixation and ΔH^+^ during C_3_ to CAM transition in the stressed state [26]; (5) weak CAM, with similar CO_2_ uptake pattern as strong CAM but less nocturnal acid accumulation.

**Figure 3:**
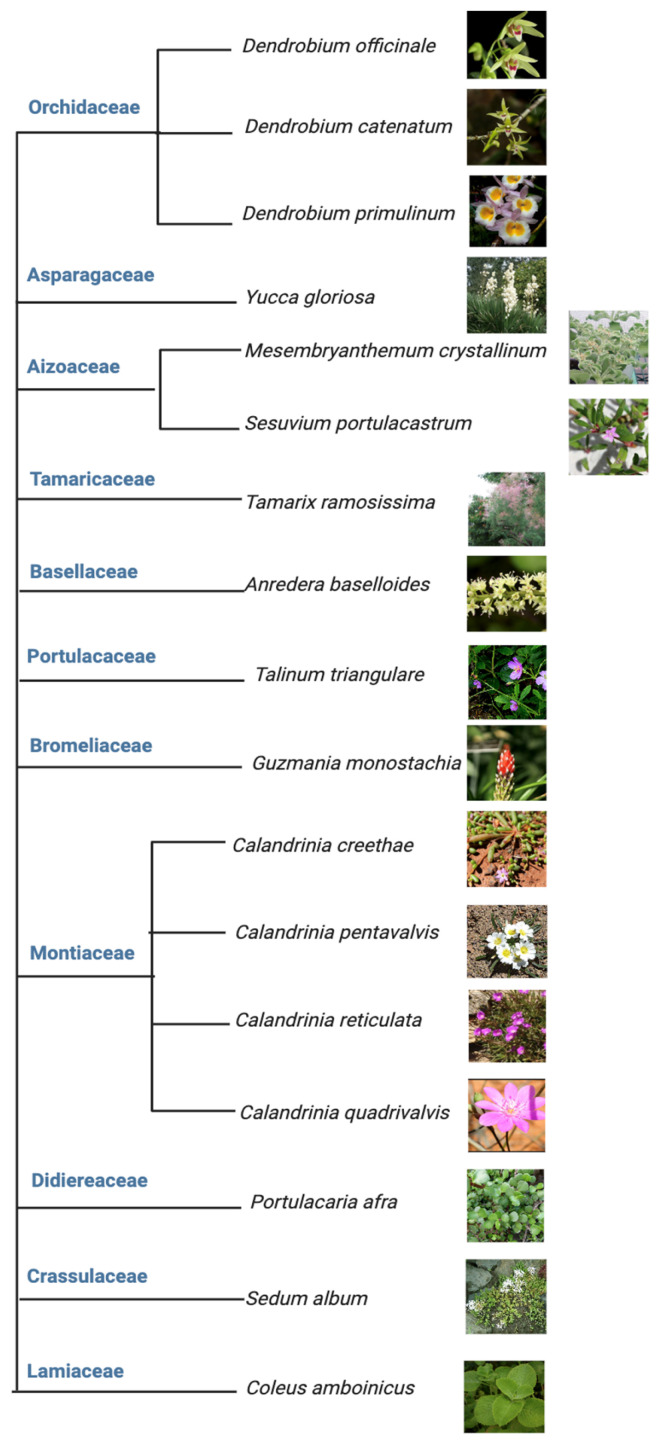
Tree view of all the facultative CAM plants investigated from 2017 to 2023. Source of each image was cited from top to bottom [38,39,40,41,42,43,44,45,46,47,48,49,50,51,52,53], respectively, except that the image of *M. crystallinum* was from the Chen lab.

Among the above five different modes of CAM, a preeminent model for elucidating the molecular underpinnings of CAM is facultative CAM [27,28]. In facultative CAM species, CAM may be induced by a variety of stimuli such as drought [29,30], salinity [19,31], high photosynthetic photon flux [6,32], abscisic acid (ABA) [33], photoperiod [34] and hydrogen peroxide [35]. Clearly, CAM plasticity is best represented by facultative CAM plants, which employ the C_3_ photosynthesis under non-stress conditions to maximize growth, but are able to undergo a gradual C_3_ to CAM transition to reduce water loss and maintain photosynthetic integrity under water-limited conditions. It ultimately translates into high WUE, survival, and reproductive success [36]. Facultative CAM plants have been identified in a wide range of plant families, such as *Bromeliaceae*, *Cactaceae*, *Aizoaceae*, *Montiaceae, Lamiaceae, Vitaceae, and Didiereaceae* [37], indicating multiple independent evolutionary events (Figure 3). Whether these independent events generated similar or different genetic and epigenetic changes that enable facultative CAM deserves immediate investigation, e.g., by identifying and utilizing the evolutionary pairs of C_3_ and CAM species.

## 3. Studies on C_3_ to CAM Transition Revealed Important Molecular Players

Over the past decades, different model species were used for diverse aspects of research pertinent to CAM. Here are six main areas of CAM research: (1) CAM ecophysiology to study and discover new CAM species in different ecological environments (e.g., [27,54]); (2) CAM origin and evolution (e.g., [55,56]); (3) genomic features and molecular mechanisms regulating CAM (e.g., [57,58]); (4) C_3_ to CAM transition (e.g., [19,20]); (5) CAM metabolic modeling (e.g., [59,60]); and (6) engineering CAM into C_3_ plants (e.g., [5,61]). All these areas of basic research aim for the ultimate goal of exploiting the potential of CAM in crop improvement under climate change [56]. Based on recent publications, *Ananas comosus* (Pineapple), *Kalanchoë fedtschenkoi*, and *Mesembryanthemum crystallinum* (Common ice plant, Table 1) are the three most extensively studied CAM models. Their genomes have been fully sequenced [58,62,63]. For studying the C_3_ to CAM transition, *M. crystallinum* has been the classic model, and *Talinum triangulare* is an emerging model [17]. *T. triangulare* is an herbaceous weed that shifts from C_3_ to CAM photosynthesis on day 11 of drought treatment. The large evenly green leaves, rapid growth, relatively short life cycle, self-cross, and full reversibility of CAM make it a model system to study facultative CAM [28].

The early research of C_3_ to CAM transition mainly focused on several key CAM enzymes, such as PEPC [68], PEPC protein kinase (PPCK) and malic enzymes, as well as metabolite transport in the C_3_ and CAM state [69,70]. Early studies suggest that ABA signaling, Ca^2+^ signaling, and protein phosphorylation/de-phosphorylation may play important roles in the C_3_ to CAM transition, whereas no key players such as kinases/phosphatases were identified. Sequence analyses were also performed on *PPC1* and *PPC2*, which are CAM-specific genes that encode PEPC [71,72]. With the emergence of microarray technologies, large-scale mRNA profiling was carried out [73]. Instead of studying the transition, Cushman group compared the gene expression of ice plants between the non-stressed C_3_ group and the induced-CAM group after 14 days of salt treatment. Gene expression of eight transporters was analyzed to study the inter-organellar metabolite transport between C_3_ and the CAM group of ice plants [74]. However, there is a lack of understanding of the temporal metabolic and molecular control of the C_3_ to CAM transition and a systems-level understanding was needed to reveal the regulatory changes underlying the transition.

With the advances in high-throughput omics technologies and computational biology, systems biology has become a prevalent approach for discovery (hypothesis generation) and functional studies (hypothesis testing). Beyond traditional physiological and biochemical methods, multi-omics (genomics, transcriptomics, proteomics and metabolomics) has generated a systems-level understanding of temporal molecular and metabolic controls underpinning CAM [75]. In *T. triangulare* leaves, targeted metabolite profiling and RNA sequencing were performed to reveal the rewiring of carbohydrate metabolism and candidate transcription factors (TFs) in the drought-induced CAM transition process [76]. Three years later, the same group identified seven candidate regulators of ABA-induced CAM including *HEAT SHOCK TF A2*, *NUCLEAR FACTOR Y*, *SUBUNIT*S *A9*, and *JmjC DOMAIN-CONTAINING PROTEIN 27* [77]. In addition to the traditional drought and salt induction of CAM, hydrogen peroxide was shown to be able to induce CAM in *M. crystallinum* [35]. In the leaves of another facultative bromeliad *Guzmania monostachia*, increases in the expression of CAM-related genes (*PEPC1*, *PPCK*, *NAD-malate dehydrogenase*, aluminum-activated malate transporter 9 (*ALMT9*), PEP carboxykinase (*PEPCK*)) and *UREASE* transcripts were shown under drought. And the role of integrating N and C metabolism of urea was suggested [78]. The CAM gene expression, antioxidant activities, and chlorophyll fluorescence were compared between a C_3_-CAM facultative species (*Sedum album*) and a C_4_-CAM facultative species (*Portulaca oleracea*) [79]. The level of nitric oxide (NO) was found to be correlated with the CAM expression during CAM induction only in *S. album* but not *P. oleracea*. This suggests the different roles of NO in C_3_ and C_4_ species during CAM induction. All the aforementioned studies did not identify a critical transition period, which is key to capturing the molecular switches for CAM. Three years ago, the transition period of *M. crystallinum* was first defined during salt-induced C_3_ to CAM shift, and further validated in independent studies through RNA-seq and physiological analyses [19,20,67]. Interestingly, three phytohormones, jasmonic acid (JA), cytokinin, and ABA were reported to play important roles in the inversed pattern of stomata opening/closing during the transition of *M. crystallinum* [67]. With the release of the ice plant genome [62], more studies can explore the genes and metabolites pertinent to the C_3_ to CAM transition.

## 4. CAM Engineering toward Solving the Global Climate Challenges

The evolution of CAM is a natural innovation in response to the hotter and drier environment. Scientists are striving to gain a better understanding of CAM associated with high WUE, and to expand the CAM characteristics to agriculturally valuable C_3_ crops. Although CAM mode compromises growth over survival, moving some of the CAM characteristics (e.g., inversed stomatal movement) into C_3_ crops without compromising yield is highly attractive and promising. Here are the rationales: (1) The existence of facultative CAM plants, such as *M. crystallinum* (see discussion in the next paragraph); (2) Unlike C_4_ photosynthesis, CAM is a single-cell phenomenon. All the genetic components, enzymes, and transporters of CAM are found in C_3_ plants [17]; (3) CAM has emerged from ancestral C_3_ photosynthesis independently in diverse plant lineages. The possible existence of multiple mechanisms presents opportunities for synthetic biology; (4) Recent omics/systems biology efforts have identified many molecular components important for the development of CAM [5,19,66,77,80]. They will certainly facilitate synthetic biology applications; (5) Chimeric/bifunctional promoters made inducible CAM a near reality. For example, guard cell-specific promoters that are also inducible by drought and switching off without drought [81,82]. The promoters will allow CAM to be turned on under adverse environmental conditions and turned off when conditions improve, so that crops become resilient and maintain productivity.

There is an ongoing debate on the C_3_ to CAM continuum. Supported by the existence of facultative CAM plants, accumulation of malate at night in C_3_ plants, and constraint-based modeling data [60], one side believes that the emergence of CAM could occur simply by increasing the pre-existing metabolic fluxes in diurnal decarboxylation from malate to CO_2_, CO_2_ re-carboxylation into the Calvin cycle, nocturnal CO_2_ carboxylation to malate, and PEP replenishment [55,60]. However, the other side believes that CAM should be regarded as a discrete metabolic innovation and argues that the theory of C_3_-CAM continuum underestimates the effort needed to activate CAM, which requires metabolic reprogramming [83]. After examining 30 CAM species and 40 C_3_ species, the authors concluded that nocturnal acidification is the hallmark of CAM. Also, although CAM plants show plasticity and have a dispersed occurrence, they only account for a small proportion (~7%) of vascular plants [83]. The ecophysiological studies involving the carbon-isotope ratio showed that the facultative CAM state is not favored [28]. The evidence seems to point to the fact that CAM is not a facile trait to develop simply by increasing the metabolic fluxes. Disentangling how CAM occurs is essential to guide the CAM engineering direction toward success.

Functional analysis of CAM-related genes not only validates the omics discovery, but also lays a solid foundation for CAM engineering. Gene function analysis has been conducted in the reference plant *Arabidopsis thaliana* by assessing 13 key CAM enzymes and regulatory proteins from the ice plant [61]. Large cell size and succulence may be needed in terms of CAM engineering due to the storage of nocturnal organic acids and water. By overexpressing a TF *VvCEB1_opt_* from *Vitis vinifera* in *Arabidopsis*, the cell size and tissue succulence were enhanced [5,84]. PEPC enzymes and their coding genes have been mostly studied in the CAM field. A partial CAM pathway was assembled by expressing an engineered *Solanum tuberosum PEPC* in *A. thaliana* under the control of a dark-induced promoter from *A. thaliana* [85]. Overexpression of *PEPC* from *Agave americana* in tobacco showed improved biomass production under stress conditions [86]. TFs play vital roles in regulating various cellular processes, and the TF-based engineering approach has the potential to enhance abiotic stress tolerance in plants [11]. Several well-known TF families including homeobox (HB), NAM, ATAF1/2, and CUC2 (NAC), WRKY, and basic region/leucine zipper motif (bZIP) are linked to abiotic stress responses. *HB7* was highly upregulated in both facultative CAM plants, *T. triangulare* [76] and *M. crystallinum* [19] in stress-induced CAM. Later functional studies showed that overexpression of the TF *McHB7* in *M. crystallinum* [80] and *A. thaliana* [87] improved plant growth and salt tolerance. An ideal proposal for future CAM synthetic biology and engineering effort would be to enhance crop WUE and resilience (through stress-inducible promoters) without negatively affecting the yield so that crops can survive the drought and heat episodes and maintain productivity.

## 5. Pressing Problems and Challenges in CAM Research

Other than being a model to study the shift from C_3_ to CAM, facultative CAM plants are also used to study the molecular mechanisms underlying stress tolerance [87,88]. As described in previous sections, *M. crystallinum* is a great model for elucidating molecular mechanisms of C_3_ to CAM transition, it is challenging to distinguish stress-related responses from CAM-related responses. For example, transcripts involved in the ABA signaling pathway and sugar metabolism showed differential expressions. But it’s challenging to know if these are stress or CAM responses. One way to overcome this dilemma is to focus on molecules/pathways that show shared changes during the CAM transition induced by different stresses, e.g., both drought and salinity [66,89,90]. These shared changes may represent evolutionary innovations for the C_3_ to CAM transition, not just stress-specific responses. Additionally, there are a number of studies that employ comparative analyses between C_3_ and CAM plants, such as comparative genomics [6], transcriptomics [91], and stomatal responses [92], which provide the static contrasts in genes, expression, regulation, and possible evolutionary mechanisms. A caveat of these studies is that phylogenetic closest pairs of C_3_ and CAM species were not identified and used. These comparisons are more likely to lead to the original molecular changes/switches for CAM evolution. Besides, the dynamic changes during the C_3_ to CAM transition are often overlooked.

Thanks to the release of genome sequencing data from obligate CAM plants, namely, *Phalaenopsis equestris* [93], pineapple [63], *K. fedtschenkoi* [58], *Carnegiea gigantea* [94], *I. taiwanensis* [12], and *Cissus rotundifolia* [95], the investigation of obligate CAM plants is considerably more comprehensive than facultative CAM plants. Paired with transcriptomics data, these works provide rich information not only on whole-genome duplication events during the CAM evolution and comparative genomics, but also on the mechanisms of how CAM operates and is modulated by studying the diel expression patterns of CAM-related genes. For example, the linkage between CAM and the circadian clock was first reported by showing that CAM genes were enriched with five circadian cis-regulatory elements (the Morning Element (CCACAC), the Evening Element (AAAATATCT), the CCA1-binding site (AAAAATCT), the G-box (CACGTG) and the TCP15-binding motif (NGGNCCCAC)) [63]. Four *K. fedtschenkoi* genes showed convergent changes in protein sequences and 60 genes showed convergent diel expression changes and convergent evolution in a variety of CAM species [58]. These results clearly documented specific components and requirements for CAM functionality. None of the model facultative CAM plants had available genome sequencing data until the first published assembly of the *M. crystallinum* genome in 2022 [62]. But the authors only sequenced the coding region and released some intermediate files which makes the genome information mostly inaccessible. Recently, the first available ice plant whole genome was released [96]. A total of 49,782 locus IDs were generated by next-generation sequencing. Further transcriptomics and proteogenomics experiments may be needed to study the expression and regulation of these identified genes.

**Figure 4:**
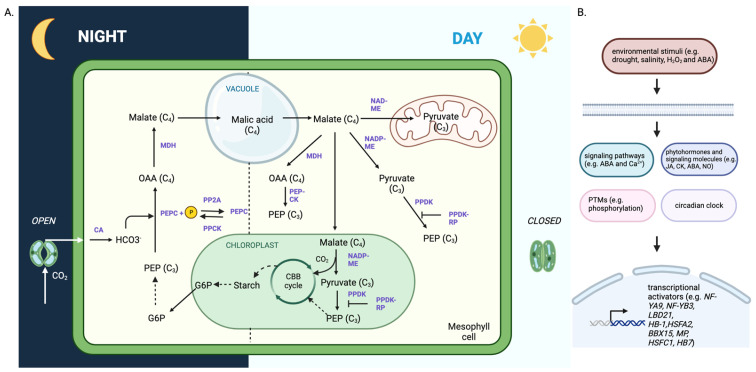
Summary of CAM diel cycle and potential CAM regulatory switches. (**A**) A simplified view of the CAM diel cycle including pathways, subcellular compartments, key intermediate metabolites, enzymes, and regulatory proteins. Solid arrow indicates a single-step process and dotted arrow indicates a multi-step process. (**B**) A diagram showing the potential regulatory switches of the C**_3_**-CAM transition. Abbreviations: CA, carbonic anhydrase; PEP, phosphosphoenolpyruvate; PEPC, PEP carboxylase; PP2A, protein phosphatase 2A; PPCK, PEPC kinase; OAA, oxaloacetate; MDH, malate dehydrogenase; NAD-ME, NAD-dependent malic enzyme; NADP-ME, NADP-dependent malic enzyme; PEP-CK, PEP carboxykinase; PPDK, pyruvate phosphate dikinase; PPDK-RP, PPDK regulatory protein; CBB cycle, Calvin-Benson-Bassham cycle; G6P, glucose 6-phosphate; H**_2_**O**_2_**, hydrogen peroxide; ABA, abscisic acid; JA, jasmonic acid; NO, nitric oxide; CK, cytokinin; PTM, post-translational modification; NF-YA9, nuclear factor Y subunit A9; NF-YB3, nuclear factor Y subunit B3; LBD21, LOB domain-containing protein 21; HB, homeobox; HSFA2, heat-inducible transcription factor A2; BBX15, B-box type zinc finger protein 15; MP, MONOPTEROS; HSFC1, heat-inducible transcription factor C1.

CAM is an intricate trait that needs not only simply integrating different functional modules, but also the tight regulation of metabolic processes. The complexity of the C_3_ to CAM transition also comes from the integration of diurnal and circadian rhythms, stomatal regulation, leaf anatomy, cell architecture, cell packing, and all the biochemical processes behind it. Different enzymes and pathways employed by different plant lineages (derived from the multiple independent evolution events) also complicate CAM engineering. Most research efforts have been committed to the carboxylation process, yet relatively little attention has been given to decarboxylation, regeneration of PEP, and energization processes. It’s known that there are two carbon breakdown pathways, phosphorolytic and hydrolytic degradation. More complicated, there are two malate decarboxylation routes, via malate dehydrogenase (MDH) and PEP-CK, and various malic enzymes including cytosolic and chloroplastic NADP-malic enzyme (NADP-ME) and mitochondrial NAD-malic enzyme (NAD-ME) (Figure 4). Despite deeming ALMT9 as the influx transporter, the efflux transporter remains unknown and the control of these two steps still needs to be addressed. Importantly, the regulatory molecular switches, including epigenetic controls, alternative splicing, non-coding RNAs, small RNAs, TFs, kinases/phosphatases, and other posttranslational modification (PTM) regulators have not fully been investigated.

## 6. Perspectives and Future Directions

Due to the limitations in genome availability, research on facultative CAM plants lags largely behind that on obligate CAM plants. Recently, genome editing tools like CRISPR/Cas9 and RNAi have been applied to two obligate CAM plants *K. fedtschenkoi* [97] and *K. laxiflora* [57]. With the availability of the genome of the classic facultative CAM model [62,96], *M. crystallinum*, more attention and research investment in facultative CAM plants and C_3_ to CAM transition could be anticipated, and it will be exciting to see the expansion of single-cell omics approaches to facultative CAM plants.

Phosphorylation is one of the ubiquitous PTMs that regulate protein functions and plant physiological output. It’s well known that PEPC activity is modulated through phosphorylation by a protein kinase PPCK. Some early papers showed that one of the decarboxylation enzymes, PEP-CK, is regulated by phosphorylation [98,99]. Acetylation, nitrosylation, and phosphorylation have been identified in NAD-ME and NADP-ME [17]. PTMs could be investigated to close the knowledge gap in facultative CAM, especially those important in triggering the transition from C_3_ to CAM (Figure 4). Mass spectrometry is a powerful analytical tool for the discovery of proteins, metabolites, and PTMs. The sensitivity of mass spectrometers has been improved significantly toward single-cell analysis [100,101,102]. A large volume of data generated by omics experiments tend to have false positives. More functional validation studies through the community-wide effort are needed to validate the molecular components, changes, and regulations in the CAM transition process.

The computational/mathematical modeling of CAM dates back to 30 years ago [103]. The earlier type of model was the ordinary differential equations (ODE) model that simulated the CAM phenomenon by turning a simplified scheme of metabolic reactions of CAM into ODE to study diel rhythmicity [103]. With a different purpose, the flux balance analysis (FBA) model is used to model the metabolic fluxes in CAM plants under different conditions and predict the optimal flux distribution that maximizes or minimizes a specific purpose, such as biomass production, ATP synthesis, or malate accumulation, while satisfying the stoichiometric and thermodynamic constraints of the system [104]. Whereas the ODE model requires the input of quantitative data of the pathways to be modeled, such as enzyme activities, to accurately present the metabolite dynamics, the recent FBA models, on the other hand, focus on studying the metabolic steady-state flux distribution [59,60,105]. The FBA model doesn’t require the input of quantitative data but also doesn’t provide information that ODE can provide, e.g., metabolite concentrations and changes [104]. In a nutshell, ODE models offer temporal dynamics of CAM and provide a mechanistic understanding of the system but rely on high-quality data input and the models are more complicated. FBA models can be integrated with genome-scale data and excel in predicting metabolic flux distributions and metabolic phenotypes under varying environmental conditions or genetic perturbations. During these 30 years, a number of ODE and FBA models adapted from their previous generations have been improved. Better models to incorporate mesophyll conductance are to be expected [17]. With the advancement of single-cell omics [106,107,108], cell-type specific models will allow prediction of how CAM may function in response to different environmental factors at the single-cell resolution.

CAM can be induced in facultative CAM plants by different conditions. There are some comparative omics studies using different plant lineages. But there have not been any studies to compare the different treatments using the same species. For example, comparative studies may be performed on the signaling components under scrutiny to show if there are any shared pathways among the different treatments of inducing CAM, such as drought, salinity, or ABA. With the advancement of cutting-edge technologies, such as single-cell analysis, artificial intelligence in plant biology, and synthetic biology, the development of cell-type specific and stress-inducible CAM in major C_3_ crops (e.g., soybean, cotton, and alfalfa) is within sight.

## 7. Concluding Remarks

As the global population is projected to reach 9 billion in 27 years, food production has become increasingly limited due to the demanding crop irrigation and the increasingly frequent drought episodes driven by climate change. CAM is a natural innovation for high WUE and stress resilience. In the era of system biology and synthetic biology, engineering the CAM characteristics into C_3_ (or C_4_) crops represents a potential breakthrough for meeting the global challenges of population growth and food security. The CAM research areas covered in this review are not mutually exclusive. Instead, they inform each other and deepen our comprehension of the evolutionary and molecular underpinnings of CAM. The C_3_-CAM transition will provide advantages to plants in the following aspects: 1. water-use efficiency 2. drought tolerance 3. temperature resilience 4. plasticity in photosynthesis 5. carbon storage. Studying the C_3_ to CAM transition using facultative CAM plants allows going back to evolution history and identifying the molecular switches (e.g., TFs and kinases) essential for the development of CAM characteristics. Once we know the “codes” for the CAM characteristics, we can use the synthetic biology “language” to build cell-type-specific circuitry for enabling important C_3_ crops with enhanced WUE, stress resilience, and improved yield.

## Figures and Tables

**Table 1:** Research progress on CAM in *M. crystallinum* from 2017 to 2022.

Research Focus	Key Findings	Reference
Identified C_3_-CAM transition period and temporal physiological changes	- The shift in a 3-day period - CO_2_ exchange reflects inversed stomatal behavior - A Python script was created for high throughput leaf area assay	[20]
Transcriptomics of guard cells during the C_3_-CAM transition	- 18 transcription factors identified	[19]
	- Guard cell has its own transition	
Nocturnal carboxylation is coordinated with starch degradation by the products of these pathways, such as carbohydrates	- Transitory starch is necessary for CAM operation - Carbohydrates coordinate the regulation of carboxylation and starch degradation	[64]
Functional CAM withdrawal in the de-salted plants	- Rapid downregulation of *PEPC1* and decrease in Δ malate was found - CAM-specific antioxidative enzyme activities exhibited transient & fully reversible responses to salt stress	[65]
Comparative proteomic changes in guard cells and mesophyll cells during the C_3_-CAM transition	- Guard cells and mesophyll cells showed different changes in proteome during the transition - Proteins involved in osmotic adjustment, ion transport, energy metabolism and light response may play important roles in the transition	[66]
Phytohormones in the stomatal behavior during the C_3_-CAM transition	- Diurnal balance of cytokinin and jasmonic acid regulates stomatal behavior	[67]
Genome sequencing, transcriptomics, and comparative genomics of leaves	- Chromosome rearrangements and gene loss in ice plant evolution - Several key CAM-related genes identified	[62]

Note: Δ malate represents the difference in malate concentration between the end of the night period and the beginning of the light phase.

## Data Availability

Not applicable.
